# Effective Biofilm Eradication on Orthopedic Implants with Methylene Blue Based Antimicrobial Photodynamic Therapy In Vitro

**DOI:** 10.3390/antibiotics12010118

**Published:** 2023-01-08

**Authors:** Julia Prinz, Marianne Wink, Sonja Neuhaus, Markus C. Grob, Heinrich Walt, Philipp P. Bosshard, Yvonne Achermann

**Affiliations:** 1 Department of Dermatology, University Hospital Zurich, University of Zurich, 8091 Zurich, Switzerland; 2 Institute of Polymer Nanotechnology, University of Applied Sciences and Arts Northwestern Switzerland, 5210 Windisch, Switzerland; 3 Institute of Polymer Engineering, University of Applied Sciences and Arts Northwestern Switzerland, 5210 Windisch, Switzerland; 4 Department for Cranio-Maxillofacial Surgery, University Hospital Zurich, University of Zurich, 8091 Zurich, Switzerland; 5 Department of Internal Medicine, Hospital Zollikerberg, 8125 Zollikerberg, Switzerland

**Keywords:** photodynamic therapy, methylene blue, biofilm, orthopedic implants, periprosthetic joint infection, in vitro

## Abstract

Periprosthetic joint infections (PJI) are difficult to treat due to biofilm formation on implant surfaces, often requiring removal or exchange of prostheses along with long-lasting antibiotic treatment. This in vitro study investigated the effect of methylene blue photodynamic therapy (MB-PDT) on PJI-causing biofilms on different implant materials. MB-PDT (664 nm LED, 15 J/cm^2^) was tested on different *Staphylococcus aureus, Staphylococcus epidermidis*, *Escherichia coli* and *Cutibacterium acnes* strains in both planktonic form and grown in early and mature biofilms on prosthetic materials (polyethylene, titanium alloys, cobalt–chrome-based alloys, and bone cement). The minimum bactericidal concentration with 100% killing (MBC_100%_) was determined. Chemical and topographical alterations were investigated on the prosthesis surfaces after MB-PDT. Results showed a MBC_100%_ of 0.5–5 μg/mL for planktonic bacteria and 50–100 μg/mL for bacteria in biofilms—independent of the tested strain, the orthopedic material, or the maturity of the biofilm. Material testing showed no relevant surface modification. MB-PDT effectively eradicated common PJI pathogens on arthroplasty materials without damage to the materials, suggesting that MB-PDT could be used as a novel treatment method, replacing current, more invasive approaches and potentially shortening the antibiotic treatment in PJI. This would improve quality of life and reduce morbidity, mortality, and high health-care costs.

## 1. Introduction

The increasing use of orthopedic implants has led to a rise in periprosthetic joint infections (PJI) [1]. The same bacteria that colonize the skin surface can contaminate the orthopedic implant and lead to PJI, with the most commonly culprits being staphylococci, streptococci and anaerobic bacteria [2]. Being one of the feared complications after implanting an orthopedic device, PJI occur despite standard preoperative skin antisepsis with alcohol-based solutions and antibiotic prophylaxis [3,4]. Hence, current preventions strategies are ineffective. Treatment of PJI is challenging due to biofilm formation on the implant surface and in the periprosthetic tissue. In biofilms, the bacteria are embedded in a matrix of extracellular polymeric substances—at least partially produced by the bacteria themselves—which helps protect them against the body’s immune system and antibiotic treatments [5]. Biofilms are defined as sessile communities of microbial cells whose formation is a cyclic process of attachment, maturation, and dispersal involving various genes [6]. The degree and rate of bacterial attachment to various materials depends on the bacterial cell surface as well as on the roughness, physiochemical properties, and hydrophobicity of the material [7]. In experimental settings, as few as 100–1000 bacteria were able to form a new biofilm on implants [8]. Therefore, high killing efficacy is required, i.e., fewer than 100 residual actively replicating planktonic bacteria, as well as bacteria within a mature (late stage) biofilm. In addition, an extensive surgical debridement of the infected tissue and a prolonged antibiotic treatment of 6 to 12 weeks is needed for clinical success in patients with an infection [9,10]. Novel treatment strategies that allow the prosthesis to be preserved and reduce the length of antibiotic treatment would be highly advantageous.

Photodynamic therapy (PDT) is a well known technique that is successfully used in dermatology and oncology and is gaining interest for the prevention and treatment of drug-resistant bacterial infections [11]. PDT, which destroys pathogens via several mechanisms, uses a light-active substance, called a photosensitizer, that is activated by light of a spectral wavelength. In the presence of oxygen, the excited photosensitizer transfers electrons to organic substrates, producing reactive oxygen species (type I reactions) or transfers energy to molecular oxygen, leading to singlet oxygen production (type II reactions), both of which cause oxidative stress on bacterial cells [12,13]. Additionally, a type III reaction mechanism is possible by which the excited photosensitizer directly targets nucleic acids, proteins, or other bio-macromolecules, independent of oxygen [14]. PDT offers several advantages over antibiotics, including the non-pharmacological effect of the photosensitizer unless it is excited with light of a specific wavelength, multi-target ability of the generated reactive oxygen species (singlet oxygen reacts with proteins, nucleotides and lipids), immediate microbiological effect, and lack of resistance generation following repeated exposures to the therapy [15,16,17,18,19,20]. Microorganisms are more susceptible to PDT than mammalian cells [21,22]. Given the major global threat of antibiotic resistance, PDT is especially interesting as a method of killing pathogens, including biofilm bacteria that are tolerant to antibiotics, without increasing antibiotic resistance [23,24].

Methylene blue (MB), a well known cationic phenothiazinium dye that has been on the market for a long time, is one of the most widely used and well characterized photosensitizers. It has the maximum absorption at 664 nm and can react by both type I and type II reaction mechanisms [25]. MB-PDT has shown high effectivity in nasal decolonization of *Staphylococcus aureus,* leading to significant reduction of surgical site infections [26] and recently also against SARS-CoV-2 [27,28]. It is further used in many clinical procedures, with low risk to patients [29]. MB-PDT studies on biofilms formed on commonly used orthopedic materials, such as polyethylene, titanium alloys, and cobalt–chrome-based alloys or bone cement, remain scarce, with only few studies reporting results on titanium discs [30,31] and none reporting effects on *Cutibacterium* biofilms.

This study aimed to investigate the killing effect of MB-PDT on common PJI-causing bacteria, first in their actively replicating planktonic form and then in biofilms formed on different orthopedic materials. We sought to not only achieve a bactericidal effect, i.e., 3 log_10_ reduction of viable bacteria, but rather to kill 100% bacteria with PDT to avoid regrowth and the risk of persisting infection. Thus, we defined minimum bactericidal concentrations with 100% killing (MBC_100%_) that result in no regrowth after reincubating the treated sample in fresh medium. We further investigated chemical and topographical alterations after PDT on the prosthesis surface of the most common used materials in arthroplasty.

## 2. Results

### 2.1. Planktonic Assays

We first tested our experimental setting with MB-PDT on a *S. aureus* clinical isolate in planktonic form using different MB concentrations and determined the MBC_100%_ without regrowth at 5 μg/mL with a 6 log_10_ reduction (Supplementary Figure S1, Table S1). For *Escherichia coli*, *Staphylococcus epidermidis* and *Cutibacterium acnes* clinical isolates, 0.5–1 μg/mL MB was required to reach the MBC_100%_ in planktonic assays. Light only or MB only (dark toxicity) was not bactericidal. By further comparing the results of *S. aureus* and *E. coli* ATCC strains, we found no strain dependence (Supplementary Figure S2a,b).

### 2.2. Biofilm Assays

#### 2.2.1. Biofilm Visualization

**Figure 1:**
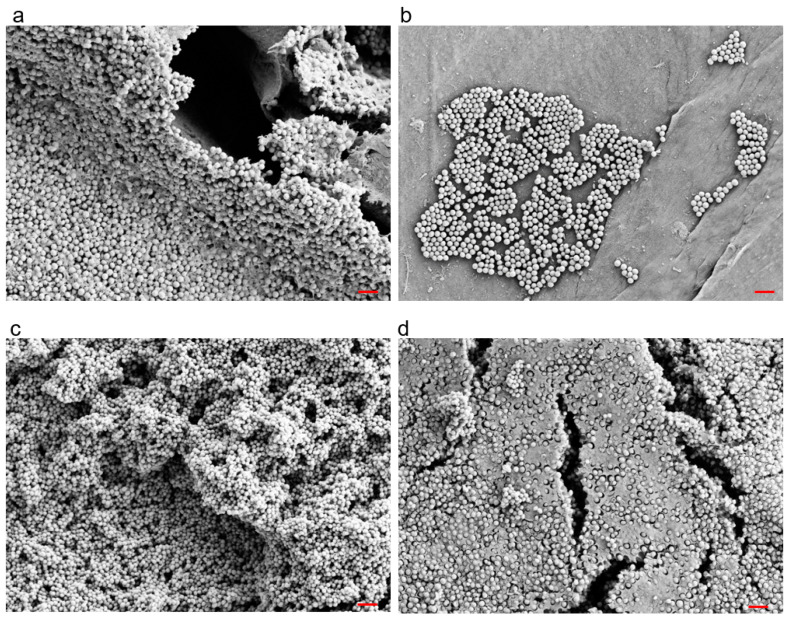
Example scanning electron micrographs of 2-day-old (**a**,**b**) and 6-day-old (**c**,**d**) *Staphylococcus aureus* biofilms grown on polyethylene discs. Red Bar = 3 μm.

To validate the experimental biofilms, we used scanning electron microscopy to visually confirm the growth of early (2-day-old) and mature (6-day-old) *S. aureus* biofilms on polyethylene (PE) discs (Figure 1). As expected, we observed that the biofilms were heterogeneously distributed on the surface and that the bacteria of the six-day-old biofilms were embedded and hidden in a thick extracellular matrix. To estimate the biofilm thickness, the difference between the top of the biofilm and the bottom of the implant disc surface was calculated. Median values showed about 20 times thicker biofilms after 6-days as compared to the 2-days (Supplementary Figure S3).

#### 2.2.2. Biofilm In Vitro Assays with *S. aureus* on Different Orthopedic Materials

**Figure 2:**
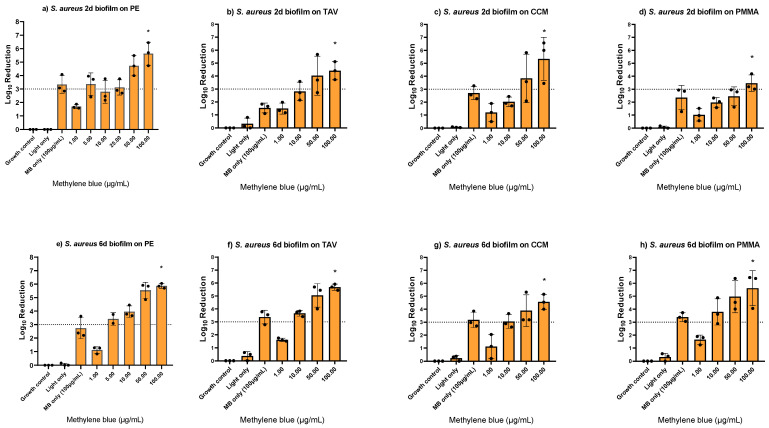
Photodynamic inactivation of *Staphylococcus aureus* biofilms formed on polyethylene (PE), titanium alloy (TAV), cobalt-chromium-molybdenum (CCM), and polymethyl methacrylate (PMMA) based bone cement discs using different concentrations of methylene blue (MB). Panels (**a**–**d**) show two-day-old biofilms, panels (**e**–**h**) show six-day-old biofilms. The bars show the average log_10_ reduction with the standard deviation from two or three independent biological replicates. The dotted line signals a bactericidal effect (3 log_10_ reductions). The stars (*) above the bars indicate 100% killing, i.e., no regrowth. MB only controls were performed with the determined minimum bactericidal concentration without regrowth (MBC_100%_).

After confirming biofilm growth on the implant discs, the MB-PDT effect was tested on early (2-day-old) and mature (6-day-old) *S. aureus* biofilms formed on different implant materials (i.e., PE, titanium alloy (TAV), cobalt-chromium-molybdenum (CCM) and polymethyl methacrylate (PMMA) based bone cement). A MBC_100%_ of 100 μg/mL MB was determined on all tested materials, with up to 5.9 log_10_ reductions independent of the biofilm thickness (Figure 2, Supplementary Table S2). Light only had no effect, while MB only at 100 μg/mL showed log_10_ reductions up to 3.4. When comparing to an early *S. aureus* biofilm of an ATCC strain, we found a lower MBC_100%_ of 50 μg/mL MB (Supplementary Figure S2c).

#### 2.2.3. Biofilm In Vitro Assays with Different Bacterial Species

**Figure 3:**
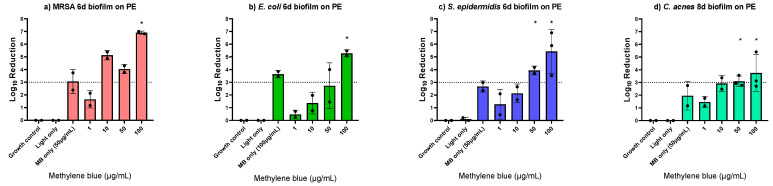
Photodynamic inactivation of six-day-old biofilms of methicillin resistant *Staphylococcus aureus* (**a**), *Escherichia coli* (**b**), *Staphylococcus epidermidis* (**c**), and *Cutibacterium acnes* (**d**) formed on polyethylene (PE) discs using different concentrations of methylene blue (MB). The bars show the average log_10_ reduction with the standard deviation from two or three independent biological replicates. The dotted line signals a bactericidal effect (3 log_10_ reductions). The stars (*) above the bars indicate 100% killing, i.e., no regrowth. MB only controls were performed with the determined minimum bactericidal concentration without regrowth (MBC_100%_).

Since the PDT killing efficacy on early and mature biofilms and biofilms on different implant materials was consistent for *S. aureus*, the MB-PDT effect was further evaluated only on PE with mature biofilms of other bacteria involved in PJI, i.e., methicillin resistant *S. aureus* (MRSA), *E. coli*, *S. epidermidis* and *C. acnes*. We found the same MBC_100%_ of 100 μg/mL MB for MRSA and *E. coli* eradication, while 50 μg/mL was enough for complete *S. epidermidis* and *C. acnes* eradication. Light only showed no effect, and MB only showed log_10_ reductions of up to 3.6 (Figure 3, Supplementary Table S3).

### 2.3. Interaction of PDT with Orthopedic Implants

None of the material testing experiments showed any alterations or damage to the materials after PDT. The attenuated total reflection infrared spectra of the different albumin-coated implant materials treated with MB-PDT with light doses from 7.5 to 75 J/cm^2^ revealed no differences between the reference and the treated samples, suggesting no signs of oxidation of the materials due to the treatment (Supplementary Figure S4). Likewise, the roughness values were comparable to the untreated reference materials for all applied light doses (Supplementary Figure S5). The confocal laser scanning microscopy (CLSM) pictures showed no visual cracks or damages on the materials after MB-PDT with the light dose of 15 J/cm^2^ (Supplementary Figure S6).

## 3. Discussion

Improved treatment outcomes in PJI may avoid the exchange of the prosthesis, leading to lower morbidity, lower mortality, and lower health-care costs. We used MB-PDT with a red-light LED at 664 nm and determined the lowest photosensitizer concentration needed for 100% killing without regrowth, i.e., the MBC_100%_. A 100% killing of bacteria in both planktonic state and in biofilms without regrowth was achieved using 0.5–5 μg/mL MB for planktonic or 50–100 μg/mL MB for early and mature biofilms of both Gram-positive and Gram-negative bacteria. PDT did not cause any relevant surface modification on different prosthesis materials suggesting that the therapy does not harm the material and is safe to use in vivo. This study demonstrates, for the first time, the eradication potential of MB-PDT on biofilms grown on different implant materials for treatment of PJI.

We found a species-dependent effect of MB-PDT in our study. In planktonic assays, the *S. aureus* MBC_100%_ was five to ten times higher compared to the other tested species—independent of the strain. Strain dependence in photodynamic inactivation efficacy has been described for clinical isolates of *S. aureus* with MB and other photosensitizers [32,33,34,35,36]. Therefore, we initially tested different strains of the same species, but since our results with ATCC strains and clinical isolates showed identical MBC_100%_, we then focused on the generally more difficult to treat clinical isolates. 

Our results showed that biofilm eradication compared to planktonic killing required 20–100 times higher MB concentrations, depending on the bacterial species. This is not unlike antibiotics, where up to 1000 times higher concentrations are required for biofilms [37,38] in order to kill commonly present bacteria tolerant to antibiotics [39,40]. In biofilm assays, *S. aureus* and *E. coli* required double the concentration compared to *S. epidermidis* or *C. acnes* isolates. However, we achieved complete eradication of all species.

Overall, the MB-PDT concentrations that we determined as MBC_100%_ to kill planktonic and biofilm bacteria were lower as compared to previous studies [30,41]. This might be due to the composition of the MB-based photosensitizer containing 0.25% chlorhexidine gluconate, which provides a booster effect on MB. Chlorhexidine is a slow release compound commonly used at 4% concentration as a skin antiseptic prior to surgery [42].

Our results confirm the high killing potential of MB-PDT on both Gram-positive and Gram-negative bacteria involved in PJI, unlike previous studies that stated difficulty killing Gram-negatives [43,44]. We achieved full eradication (up to 6 log_10_ reductions) of early and mature biofilms independent of the implant material and biofilm thickness, suggesting that MB-PDT can effectively kill PJI-causing biofilms, i.e., *S. aureus*, *S. epidermidis*, *E. coli* and *C. acnes* biofilms, on various orthopedic implant materials despite different cell surface characteristics. Few studies so far have reported eradication on titanium alloy discs [30,31] or bactericidal effects on acrylic resin discs [45].

The main limitations of this study are that the PDT experiments were only performed on static biofilms in vitro and that the toxicity of MB on human cell lines was not investigated. However, there are insufficient free-radical defence and repair mechanisms in monolayers in cell-culture assays as compared to intact tissue in vivo, hence toxicity is expected when MB is activated in monolayers. Several in vivo studies showed that MB was safe and remained without adverse events even when used in higher concentrations of 10 mg/mL [26,46,47,48]. Clinical studies report no adverse events when using MB-PDT for treatment of onychomycosis, oral candidiasis, infectious diabetic foot ulcers [48], infected wounds [49], or when used for nasal decolonization to prevent surgical site infections [26]. As a strength of this study, we used biofilm-forming clinical isolates derived from PJI patients to better mimic the in vivo situation.

To conclude, we proved in vitro the potential of MB-PDT by showing 100% killing of the most PJI-causing bacteria without harm to the prosthetic materials, independent of the orthopedic material or the maturity of the biofilm. PDT offers many advantages—i.e., immediate, broad-spectrum action after light activation of the photosensitizer, that it can be repeatedly performed, and that resistance is less likely since various biomolecules are targeted [24,50]—and a number of potential opportunities. MB-PDT in clinical practice could improve the cure rate in patients with a chronic PJI with a less invasive surgical treatment compared to patients without PDT. Besides, PDT as a novel treatment method to kill biofilm-forming bacteria in PJIs would obviate the exchange of the prosthesis and shorten the antibiotic treatment duration.

In a next step, we plan a clinical study investigating the safety of the treatment. This study may have an impact as a model for applications in other implant-associated infections. Future studies will have to show whether PDT could be added to the treatment regimen or included in a prevention strategy in which PDT is intraoperatively applied at the time of implantation to eradicate bacterial contaminants. Finally, within the discussions of antimicrobial resistance, we are convinced that further research in the field of PDT is very important to save antibiotics and thus prevent further rise of antibiotic resistance.

## 4. Materials and Methods

### 4.1. Bacterial Strains and Cultivation

The following clinical isolates from patients suffering from PJI were used for PDT experiments: *Staphylococcus aureus* (CI175), *Staphylococcus epidermidis* (CI195), *Escherichia coli* (BCI0413, provided by the Institute of Medical Microbiology, Zurich, Switzerland), *Cutibacterium acnes* (Y105). To show strain independence in treating *S. aureus*, we tested two sequenced strains—a methicillin resistant *Staphylococcus aureus* (MRSA) strain JE2 and a methicillin susceptible reference strain (ATCC 29213). For *E. coli*, the reference strain ATCC 25922 was used. The bacterial biobank was approved by the institutional review board in Zurich, Switzerland (KEK Nr 2016-00145, KEK Nr 2017-01458).

To obtain fresh cultures, *S. aureus*, *S. epidermidis,* and *E. coli* strains were grown from frozen cultures on brain—heart infusion (BHI, Becton Dickinson, Heidelberg, Germany) agar plates for 24 h at 37 °C under aerobic conditions, and *C. acnes* was grown on BHI for 3–5 days at 37 °C under anaerobic conditions (GENbags, bioMérieux, Mary-l’Etoile, France). Liquid cultures from colonies on agar plates were performed in 10 mL BHI broth at 37 °C with shaking overnight for *S. aureus*, *S. epidermidis* and *E. coli* under aerobic conditions and for three days for *C. acnes* under anaerobic conditions. Then, 1:10 dilutions in fresh media were performed and incubated for 2 h (*S. aureus*, *S. epidermidis* and *E. coli*) to obtain bacteria in their exponential growth phase. Finally, the optical density at 600 nm (OD_600_) was adjusted to 0.4 for *E. coli* or 0.5 for *S. aureus*, *S. epidermidis* and *C. acnes* to reach 10^8^ colony forming units (CFU) per milliliter as start inoculum for PDT experiments.

### 4.2. Photosensitizer and PDT Conditions

Methylene blue-based photosensitizer NF-031 was provided by Ondine Biomedical Inc. (Vancouver, Canada) with a concentration of 100 μg/mL. Dilutions were prepared in dH_2_O and kept in the dark at all times. MB was tested in concentrations from 0.25 to 100 μg/mL. The incubation time with bacteria was 10 min. Afterwards, light treatment was performed using an LED light source—provided by Ondine Biomedical Inc. (Vancouver, BC, Canada)—with a wavelength of 664 nm and a radiant fluence of 15 J/cm^2^. The homogeneity of the light source was confirmed with planktonic assays and varied 19% throughout a 24-well plate.

### 4.3. Planktonic Assays

For planktonic assays, 1 mL of the start inoculum was transferred into the wells of a 24-well plate. The cells were centrifuged (4000 rpm, 10 min) and resuspended in 1 mL photosensitizer solutions. After 10 min incubation, the cells were centrifuged and washed in dH_2_O and light treatment was performed. After PDT, the number of remaining bacteria, i.e., colony forming units (CFU), was evaluated using the colony counting method. Ten-fold serial dilutions were prepared and 10 µL thereof were spotted in triplicates on BHI agar plates and incubated for 24 h under aerobic conditions for *S. aureus*, *S. epidermidis* and *E. coli* or for 3 days at 37 °C under anaerobic conditions for *C. acnes*. The bacterial killing effect was determined as log_10_ reduction relative to the non-treated growth control. A log_10_ reduction >3 is defined as bactericidal, meaning that >99.9% of the total number of bacteria has been killed [51]. In order to determine the minimum bactericidal concentration with 100% killing (MBC_100%_), i.e., no regrowth, 50 µl of the treated samples were incubated in 5 mL fresh BHI broth at 37 °C for 2 days (*S. aureus*, *S. epidermidis*, *E. coli*) or 3 days (*C. acnes*) and subsequently subcultured on BHI agar plates for 2 days (*S. aureus*, *S. epidermidis*, *E. coli*) or 6 days (*C. acnes*). Bacteria without PDT treatment served as growth control in each experiment. As sterility controls, BHI medium and BHI medium treated with PDT were used in each experiment. Additionally, the dark toxicity of the photosensitizer (MB only) and light only controls were tested.

### 4.4. Biofilm Assays

For biofilm assays, static biofilms were grown on sterilized orthopedic implant materials (round discs of 10 mm in diameter and 1 mm in thickness) as attachment surfaces, i.e., ultra-high molecular weight polyethylene (PE), titanium alloy Ti-6Al-4V (TAV), cobalt-chromium-molybdenum (CCM) (provided by Synthes GmbH, Zuchwil, Switzerland), and polymethyl methacrylate (PMMA)-based bone cement (provided by Heraeus Group, Hanau, Germany). An amount of 2 mL of the starting inoculum were incubated with the discs in a 24-well plate and incubated at 37 °C for 2 days for early and 6 days for mature biofilm formation (*S. aureus*, *S. epidermidis*, *E. coli*), and 8 days for mature biofilm formation of *C. acnes*. The medium was regularly changed for growth of mature biofilms. Then, discs were washed three times with PBS to remove any planktonic bacteria before adding 1 mL of photosensitizer solution. After 10 min incubation, the photosensitizer was removed, and light treatment was performed on both sides of the discs with conditions as described above. The implant discs were sonicated to dissolve the biofilm bacteria using an ultrasound bath for one minute at 40 Hertz [52,53]. The colony counting and regrowth method was performed as outlined for planktonic bacteria. Biofilm bacteria grown on discs without PDT were used as growth control. As sterility controls, BHI medium only, BHI medium with discs, and BHI medium with discs and PDT, all without bacteria, were used in each experiment.

The clinical isolate *S. aureus* CI175 was used to establish the PDT protocol and was tested on all materials. Other bacteria were only tested as mature biofilms on PE. All PDT experiments were performed in biological triplicates (for *S. aureus*) or duplicates (for all other tested bacteria).

### 4.5. Biofilm Imaging

Biofilm images were performed to ensure early and mature biofilm formation on the used orthopedic materials. Biofilms were grown on PE discs for 2 days and 6 days as outlined above, washed, and immediately fixed with 2.5% glutaraldehyde in 0.1 M cacodylate buffer for 2 h (solutions provided by the Center for Microscopy and Image Analysis at the University of Zurich, Zurich, Switzerland). The samples were delivered to the Center for Microscopy and Image Analysis at the University of Zurich, Switzerland for further processing. The samples were rinsed 3 times with PBS, postfixed with 1% osmium tetroxide/ PBS (1:2) for 30 min, rinsed again 3 times with PBS, then dehydrated in 70% and 100% ethanol and hexamethyldilisazane for 1 h each prior to air drying. The samples were mounted on aluminium stubs using double-sided conductive tape and/or conductive carbon before sputter coating in a CCU-10 HV (Safematic GmbH, Zizers, Switzerland) with 4 nm of platinum (working distance 5 cm, argon pressure 0.1 mbar, sputter current 30 mA) and imaged using a GeminiSEM 450 scanning electron microscope (Zeiss, Oberkochen, Germany). Additionally, the thickness of the biofilm was estimated using working distances. Therefore, the difference between the top of the biofilm and the surface of the implant discs was calculated.

### 4.6. Investigation of Possible Impact of PDT on Orthopedic Implants Material

Implant material discs (as used for PDT experiments) were used for all material testing experiments. To mimic the situation in vivo, the discs were coated with bovine serum albumin from bovine serum (≥95%, Fluka, Buchs, Switzerland). They were rinsed with distilled water and incubated in 1 mL of 10 mg/mL bovine serum albumin solution for 24 h at 4 °C. The discs were then washed three times with distilled water. Next, the albumin-coated platelets were incubated in 100 μg/mL MB solution (methylene blue hydrate, purum ≥ 97%, Sigma, Buchs, Switzerland) for 10 min and subsequently washed once with water. The discs were placed under a 660 nm LED (L660N-66-16100, Ushio, Steinhöring, Germany) and illuminated with doses of 7.5, 15, 30 or 75 J/cm^2^ on both sides. The treated discs were investigated for signs of oxidation with attenuated total reflection infrared spectroscopy using a Ge crystal (Nicolet iD7 ATR, Thermo Scientific, Basel, Switzerland) and their surfaces were examined for cracks or damages in a confocal laser scanning microscope (CLSM) (VKX1100, Keyence, Urdorf, Switzerland). Their line roughness value as roughness average (Ra) was determined from CLSM images taken at a magnification of 5×.

## Data Availability

The data presented in this study are available in the Supplementary Material.

## Supplementary Materials

The following supporting information can be downloaded at: https://www.mdpi.com/article/10.3390/antibiotics12010118/s1, Table S1: Photodynamic inactivation of planktonic *Staphylococcus aureus*, *Escherichia coli*, *Staphylococcus epidermidis* and *Cutibacterium acnes* using different concentrations of methylene blue (MB) as average log_10_ reduction of two or three independent biological replicates. The stars indicate 100% killing, i.e., no regrowth. MB only controls were performed with the determined minimum bactericidal concentration without regrowth (MBC_100%_); Table S2: Photodynamic inactivation of early (2-day-old) and mature (6-day-old) *Staphylococcus aureus* biofilms formed on polyethylene (PE), titanium alloy (TAV), cobalt-chromium-molybdenum (CCM) and polymethyl methacrylate (PMMA) based bone cement discs. The average log_10_ reductions of two or three independent biological replicates using different methylene blue (MB) concentrations are presented. The stars indicate 100% killing, i.e., no regrowth. MB only controls were performed with the determined minimum bactericidal concentration without regrowth (MBC_100%_); Table S3: Photodynamic inactivation of mature biofilms of methicillin resistant *Staphylococcus aureus* (6d), *Escherichia coli* (6d), *Staphylococcus epidermidis* (6d) and *Cutibacterium acnes* (8d) formed on polyethylene (PE) discs. The average log_10_ reductions of two or three independent biological replicates using different methylene blue (MB) concentrations are presented. The stars indicate 100% killing, i.e., no regrowth. MB only controls were performed with the determined minimum bactericidal concentration without regrowth (MBC_100%_); Figure S1: Photodynamic inactivation of planktonic *Staphylococcus aureus* (a), *Escherichia coli* (b), *Staphylococcus epidermidis* (c) and *Cutibacterium acnes* (d) using different concentrations of methylene blue (MB). The bars show the average log_10_ reduction with the standard deviation from two or three independent biological replicates. The dotted line signals a bactericidal effect (3 log_10_ reductions). The stars above the bars indicate 100% killing, i.e., no regrowth. MB only controls were performed with the determined minimum bactericidal concentration without regrowth (MBC_100%_); Figure S2: Photodynamic inactivation of planktonic ATCC *Staphylococcus aureus* (a), planktonic ATCC *Escherichia coli* (b) and 2-day-old ATCC *Staphylococcus aureus* biofilm formed on polyethylene (PE) (c) using different concentrations of methylene blue (MB). The bars show the average log_10_ reduction with the standard deviation from two independent biological replicates. The dotted line signals a bactericidal effect (3 log10 reductions). The stars above the bars indicate 100% killing, i.e., no regrowth. Photosensitizer only (PS only) controls were performed with the determined minimum bactericidal concentration without regrowth (MBC_100%_); Figure S3: Biofilm thickness of 2-day-old and 6-day-old *Staphylococcus aureus* biofilm; Figure S4: Attenuated total reflection infrared spectroscopy spectra of four different albumin-coated implant materials (PE, polyethylene; PMMA, polymethyl methacrylate; TAV, titanium alloy; CCM, cobalt-chromium-molybdenum) treated with MB-PDT using light doses ranging from 7.5 to 75 J/cm^2^. No differences between the reference and the treated samples are visible. Adsorption of albumin was only measured on the PMMA cement materials; Figure S5: Roughness average (Ra) values of four different albumin-coated implant materials (PE, polyethylene; PMMA, polymethyl methacrylate; TAV, titanium alloy; CCM, cobalt-chromium-molybdenum) treated with PDT using methylene blue as a photosensitizer and light doses ranging from 7.5 to 75 J/cm^2^ and the untreated (0 J/cm^2^) reference materials; Figure S6: Example CLSM pictures of implant discs (top: TAV, titanium alloy; bottom: CCM, cobalt-chromium-molybdenum) before PDT (left) and after PDT (right). Scale bar = 1000 µm. No cracks or damages are seen after PDT.
