# Supplementary material for: Effective Biofilm Eradication on Orthopedic Implants with Methylene Blue Based Antimicrobial Photodynamic Therapy In Vitro
Source: Antibiotics (Basel). 2023 Jan 8;12(1):118. doi: 10.3390/antibiotics12010118 (PMC9854686; doi:10.3390/antibiotics12010118)
Supplement: Supplementary file 1 [file antibiotics-12-00118-s001.zip › antibiotics-2097544-supplementary.pdf]

Supplementary Material for Manuscript: Effective biofilm eradication on orthopedic implants with methylene blue based antimicrobial photodynamic therapy *in vitro*

Table S1: Photodynamic inactivation of planktonic *Staphylococcus aureus*, *Escherichia coli*, *Staphylococcus epidermidis* and *Cutibacterium acnes* using different concentrations of methylene blue (MB) as average log_{10} reduction of two or three independent biological replicates. The stars indicate 100% killing, i.e., no regrowth. MB only controls were performed with the determined minimum bactericidal concentration without regrowth ($MBC_{100\%}$).

Log10 reduction

**Methylene blue concentration (µg/ml)**	***S. aureus***	***E. coli***	***S. epidermidis***	***C. acnes***
**0.25**	1.57	1.42	2.49	2.30
**0.50**	2.06	3.17	4.48	3.98**
**1.00**	4.11	6.19**	6.07**	5.94*
**2.50**	5.67	6.19*	6.21*	6.26*
**5.00**	5.91*	6.19*	6.07*	5.94*
**10.00**	5.98*	6.19*	6.07*	5.94*
**MB only**	2.39	2.03	2.20	1.23
**Light only**	0.15	0.03	0.00	0.24

*100% killing, i.e. no regrowth
**Minimum bactericidal concentration with 100% killing and no regrowth (=MBC100%)
Methylene blue only was tested with the concentration of the MBC100% (5 µg/ml for S. aureus, 1 µg/ml for E. coli and S. epidermidis, 0.5 µg/ml for C. acnes)

Table S2: Photodynamic inactivation of early (2-day-old) and mature (6-day-old) *Staphylococcus aureus* biofilms formed on polyethylene (PE), titanium alloy (TAV), cobalt-chromium-molybdenum (CCM) and polymethyl methacrylate (PMMA) based bone cement discs. The average log_{10} reductions of two or three independent biological replicates using different methylene blue (MB) concentrations are presented. The stars indicate 100% killing, i.e., no regrowth. MB only controls were performed with the determined minimum bactericidal concentration without regrowth ($MBC_{100\%}$).

Log10 reduction

**Methylene blue concentration (µg/ml)**	**PE**		**TAV**		**CCM**		**PMMA Cement**	
	**2d**	**6d**	**2d**	**6d**	**2d**	**6d**	**2d**	**6d**
1.00	1.70	1.12	1.50	1.61	1.20	1.12	1.02	1.65
10.00	2.80	3.96	2.82	3.66	2.03	3.05	1.98	3.79
50.00	4.73	5.54	4.03	5.04	3.83	3.89	2.46	4.97
100.00	5.62*	5.87*	4.41*	5.68*	5.34*	4.56*	3.46*	5.62*
MB only	3.34	2.73	1.54	3.37	2.70	3.18	2.35	3.39
Light only	0.00	0.05	0.31	0.37	0.06	0.22	0.07	0.31

Table S3: Photodynamic inactivation of mature biofilms of methicillin resistant *Staphylococcus aureus* (6d), *Escherichia coli* (6d), *Staphylococcus epidermidis* (6d) and *Cutibacterium acnes* (8d) formed on polyethylene (PE) discs. The average log_{10} reductions of two or three independent biological replicates using different methylene blue (MB) concentrations are presented. The stars indicate 100% killing, i.e., no regrowth. MB only controls were performed with the determined minimum bactericidal concentration without regrowth ($MBC_{100\%}$).

Log10 reduction

**Methylene blue concentration (µg/ml)**	**MRSA**	***E. coli***	***S. epidermidis***	***C. acnes***
1.00	1.66	0.48	1.28	1.46
10.00	5.13	1.37	2.14	2.93
50.00	4.04	2.73	3.94*	3.09**
100.00	6.91*	5.28*	5.44*	3.75*
MB only	3.06	3.64	2.68	1.96
Light only	0.00	0.00	0.11	0.00

MB, Methylene blue
6d biofilm for MRSA, E. coli and S. epidermidis, 8d biofilm for C. acnes
*100% killing, i.e. no regrowth
**minimum bactericidal concentration with 100% killing and no regrowth (=MBC100%)
Methylene blue only was tested with the concentration of the MBC100%

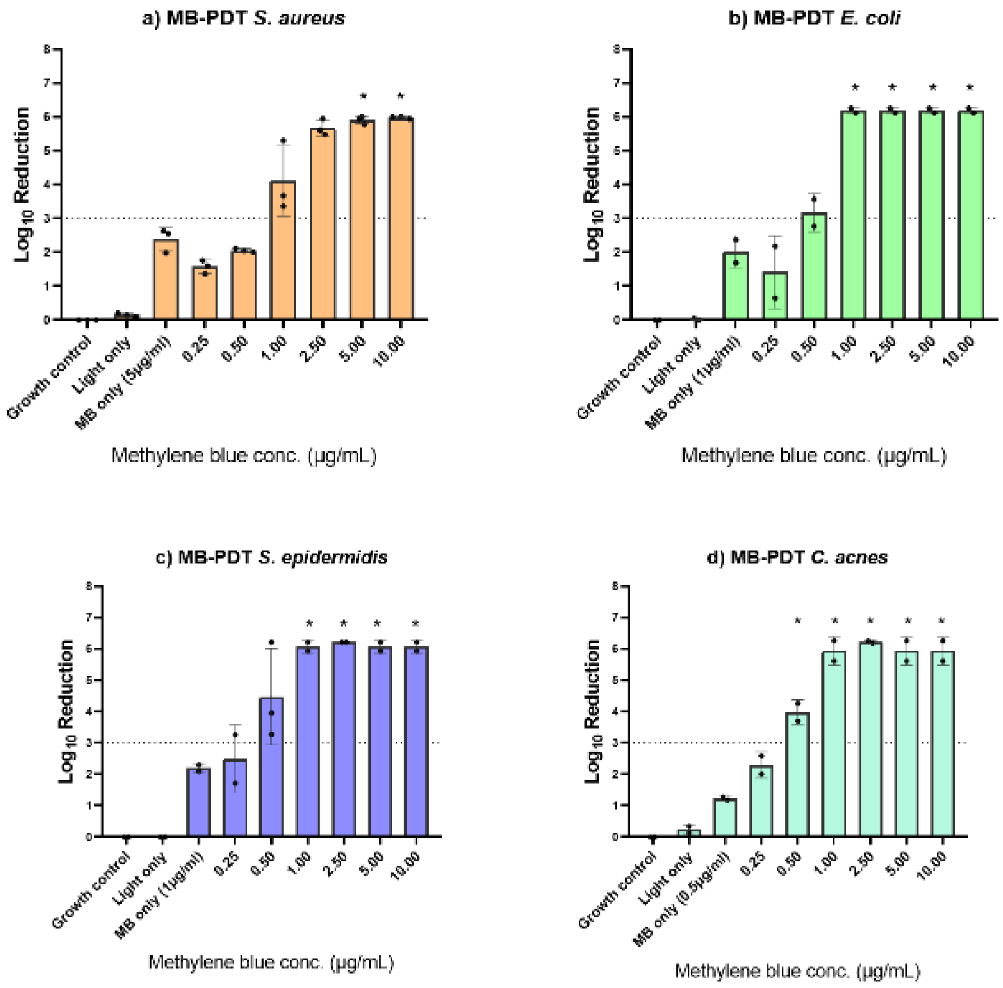

Figure S1. Photodynamic inactivation of planktonic *Staphylococcus aureus* (a), *Escherichia coli* (b), *Staphylococcus epidermidis* (c) and *Cutibacterium acnes* (d) using different concentrations of methylene blue (MB). The bars show the average log_{10} reduction with the standard deviation from two or three independent biological replicates. The dotted line signals a bactericidal effect (3 log_{10} reductions). The stars above the bars indicate 100% killing, i.e. no regrowth. MB only controls were performed with the determined minimum bactericidal concentration without regrowth ($MBC_{100\%}$).

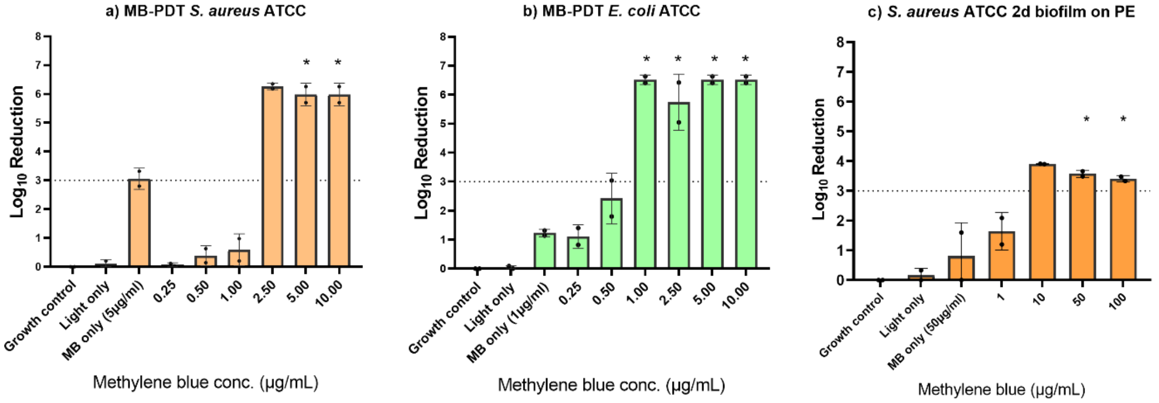

Figure S2: Photodynamic inactivation of planktonic ATCC *Staphylococcus aureus* (a), planktonic ATCC *Escherichia coli* (b) and 2-day-old ATCC *Staphylococcus aureus* biofilm formed on polyethylene (PE) (c) using different concentrations of methylene blue (MB). The bars show the average log_{10} reduction with the standard deviation from two independent biological replicates. The dotted line signals a bactericidal effect (3 log_{10} reductions). The stars above the bars indicate 100% killing, i.e. no regrowth. MB only controls were performed with the determined minimum bactericidal concentration without regrowth ($MBC_{100\%}$).

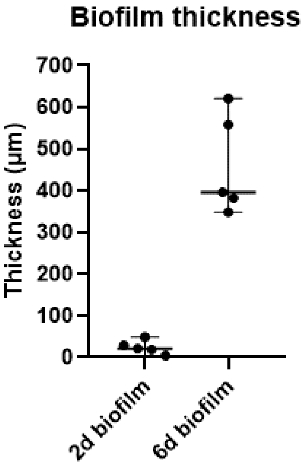

Figure S3: Biofilm thickness of 2-day-old and 6-day-old *Staphylococcus aureus* biofilm.

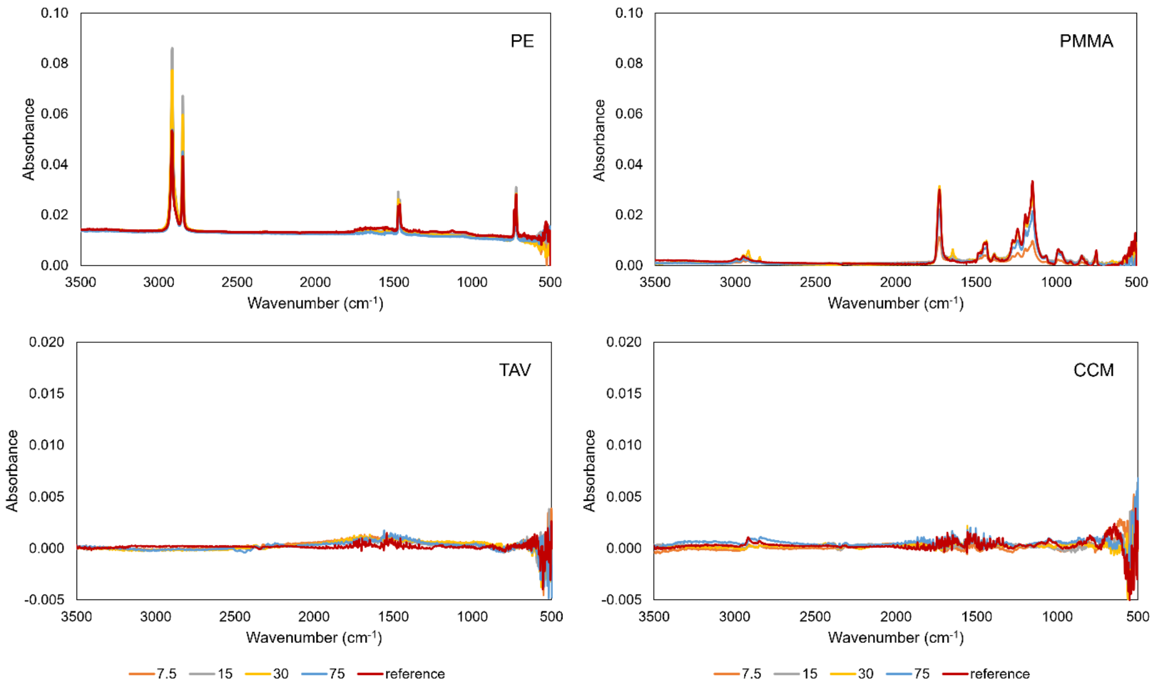

Figure S4: Attenuated total reflection infrared spectroscopy spectra of four different albumin-coated implant materials (PE, polyethylene; PMMA, polymethyl methacrylat; TAV, titanium alloy; CCM, cobalt-chromium-molybdenum) treated with methylene blue photodynamic therapy (MB-PDT) using light doses ranging from 7.5 to 75 J/cm^2. No differences between the reference and the treated samples are visible. Adsorption of albumin was only measured on the PMMA cement materials.

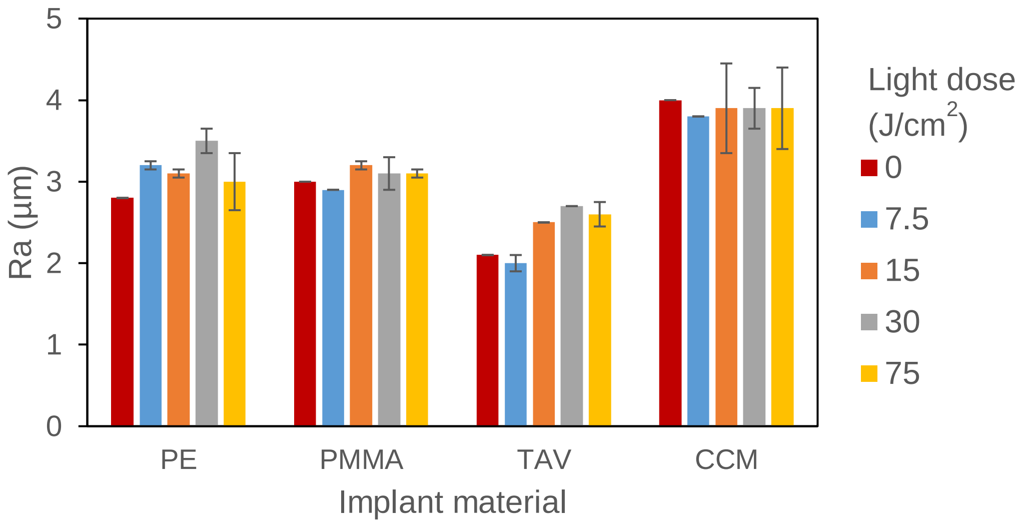

Figure S5: Roughness average (Ra) values of four different albumin-coated implant materials (PE, polyethylene; PMMA, polymethyl methacrylat; TAV, titanium alloy; CCM, Cobalt-chromium-molybdenum) treated with methylene blue photodynamic therapy (MB-PDT) using light doses ranging from 7.5 to 75 J/cm^2 compared to the untreated (0 J/cm^2) reference materials.

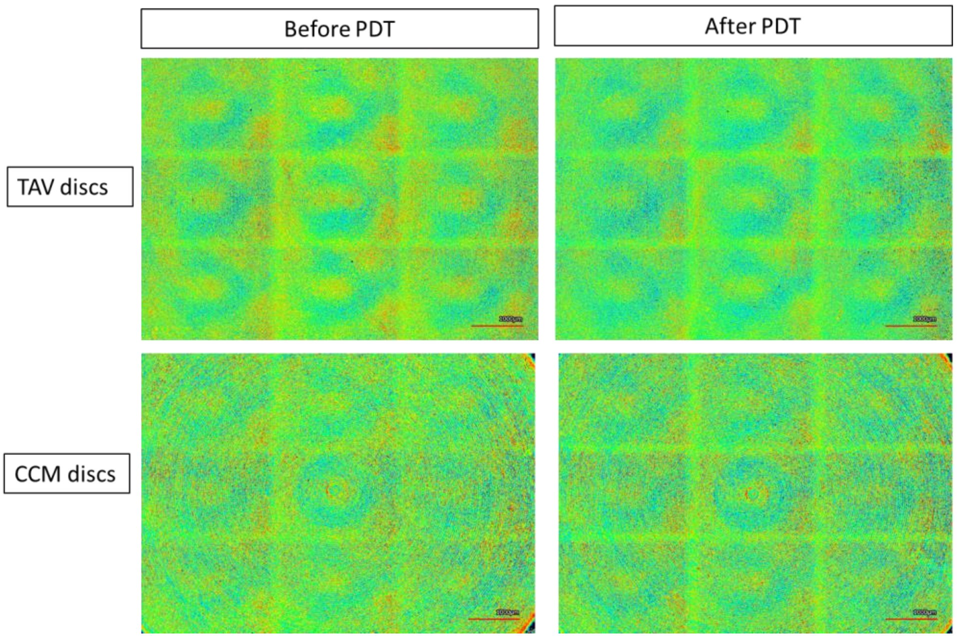

Figure S6: Example confocal laser scanning microscopy pictures of implant discs (top: TAV, titanium alloy; bottom CCM, cobalt-chromium-molybdenum) before PDT (left) and after PDT (right). Scale bar = 1000µm. No cracks or damages are visually seen after photodynamic therapy.
